# RECOLLECTING AND REFLECTING: STORY ASIDES IN YOUNG AND OLDER ADULTS’ AUTOBIOGRAPHICAL STORIES

**DOI:** 10.1093/geroni/igae098.3333

**Published:** 2024-12-31

**Authors:** Teagan McCune, Nicole Alea

**Affiliations:** University of California Santa Barbara, Santa Barbara, California, United States; University of California Santa Barbara, Santa Barbara, California, United States

## Abstract

The ways people tell autobiographical stories changes with age. One change is the amount of tangentially-related information included in these stories. These pieces of information–story asides–add context (e.g. knowledge about the world or storyteller). Story asides are expressed more frequently in the autobiographical stories of older adults compared to young adults, but the underlying mechanism is unclear. One potential mechanism is how people remember the story. The current study tested two potential mediators of this expression in young and older adults’ autobiographical stories. As part of a larger study, 61 young (M = 19.9 years) and 35 older (M = 73.8 years) adults shared an autobiographical story about the same event, a positive vacation. Participants then completed a modified version of the Autobiographical Memory Questionnaire, including 5 items about recollection (e.g., feeling the emotions they felt then) and 3 items about reflection (e.g., how significant this event is) on their autobiographical story. Mediation regression analyses indicated that story aside expression, recollecting about events, and reflecting on events all increased with increasing age. However, recollection and reflection did not predict story asides, nor were there any mediating effects of these variables between age and story aside expression in autobiographical stories. These findings suggest the way people remember and reflect on their stories is perhaps not an underlying mechanism for age group differences in story aside expression. If not the subjective indicators of memory quality and experience (recollection, reflection), then other cognitive and goal-oriented variables might explain these changes.

